# Ubiquitin Receptor RPN13 Mediates the Inhibitory Interaction of Diphenyldihaloketones CLEFMA and EF24 With the 26S Proteasome

**DOI:** 10.3389/fchem.2018.00392

**Published:** 2018-09-10

**Authors:** Geeta Rao, Gregory Nkepang, Jian Xu, Hooman Yari, Hailey Houson, Chengwen Teng, Vibhudutta Awasthi

**Affiliations:** ^1^Department of Pharmaceutical Sciences, University of Oklahoma Health Sciences Center, Oklahoma City, OK, United States; ^2^Department of Medicine, University of Oklahoma Health Sciences Center, Oklahoma City, OK, United States

**Keywords:** proteasome, CLEFMA, EF24, RPN13, proteasome inhibitors, dienone, diphenyldihaloketone

## Abstract

The proteasome is a validated target in drug discovery for diseases associated with unusual proteasomal activity. Here we report that two diphenyldihaloketones, CLEFMA and EF24, inhibit the peptidase activity of the 26S proteasome. The objective of this study was to investigate interaction of these compounds with the proteasome and identify a putative target within the protein components of the 26S proteasome. We employed standard fluorogenic peptide-based proteasome activity assay for trypsin-like, chymotrypsin-like, and caspase-like activities of human purified 26S proteasome in cell-free conditions. GFPu-1 and HUVEC cells were used as proteasome reporter cells. Direct binding studies used purified 19S, 20S, 26S, and recombinant RPN13-Pru for interaction with biotinylated analogs of CLEFMA and EF24. The reaction mixtures were subjected to horizontal gel electrophoresis, streptavidin-blotting, pull-down assays, and immunoblotting. The identity of the interacting protein was determined by 2D gel electrophoresis and LC-MS/MS. Drug affinity responsive target stability technique was utilized to examine if CLEFMA binding confers protection to RPN13 against thermolysin-catalyzed proteolysis. We found that trypsin-and chymotrypsin-like activities of the 26S proteasome were reduced significantly by both compounds. The compounds also reduced the proteolytic activity in GFPu-1 and HUVEC cells, resulting in accumulation of ubiquitinated proteins without affecting the autophagy process. From direct binding assays a 43 kDa protein in the 26S proteasome was found to be the interacting partner. This protein was identified by tandem mass spectroscopy as regulatory particle subunit 13 (RPN13), a ubiquitin receptor in the 19S regulatory particle. Furthermore, binding of CLEFMA to RPN13 did not protect latter from thermolysin-mediated proteolysis. Together, this study showed diphenyldihaloketones as potential proteasome inhibitors for treatment of diseases with perturbed proteasome function. The results also unraveled RPN13 as a unique target of CLEFMA and EF24. As a result, these compounds inhibit both trypsin-like and chymotrypsin-like proteasome activities.

## Introduction

**Figure 1:**
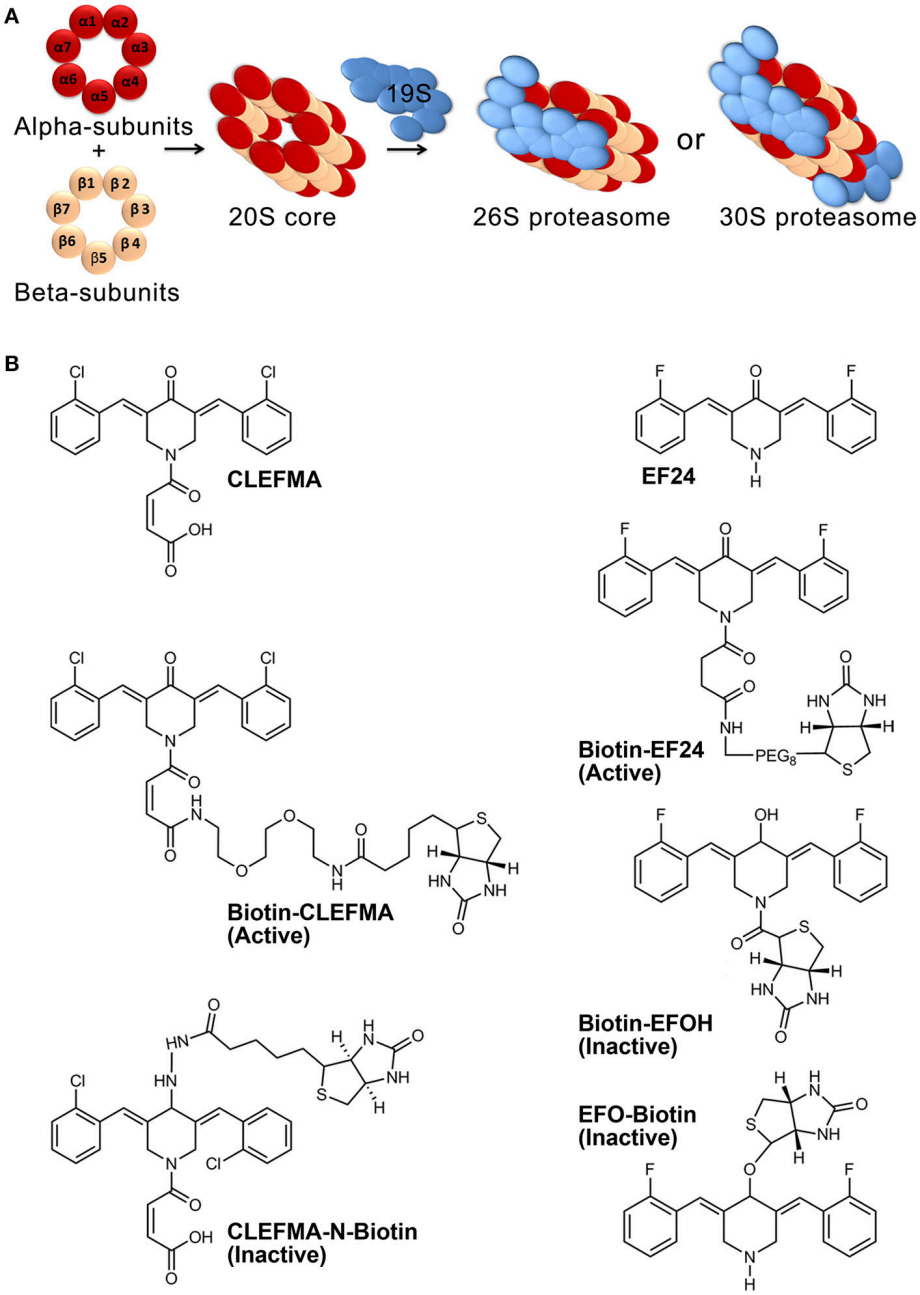
Proteasome architecture and compounds used in this work. **(A)** The 26S proteasome assembly consists of four central rings making a 20S core and single- or double-sided caps of 19S. Each of the four central rings contains seven protein subunits. **(B)** Structures of CLEFMA (4-(3, 5-bis (2-chlorobenzylidene)-4-oxo-piperidine-1-yl)-4-oxo-2-butenoic acid), EF24 (3,5-bis(2-fluorobenzylidene) piperidin-4-one), and their various biotinylated analogs.

The ubiquitin-proteasome pathway (UPP) is an intracellular protein quality control mechanism responsible for degradation of the majority of misfolded and damaged proteins in eukaryotic cells, helping maintain normal cellular proteostasis (Russell et al., 1999). In addition, it influences several critical signaling pathways by controlling the expression and function of regulatory proteins, including proteins that control inflammation, antigen presentation, cell-cycle progression, apoptosis, and DNA repair. For example, the stability and activity of HIF-1α, NF-κB, Nrf2, and p53 are primarily regulated by the UPP (Kallio et al., 1999; Villeneuve et al., 2010; Kanarek and Ben-Neriah, 2012; Love et al., 2012). Functional capacity of the UPP is attributed to a bulky multi-catalytic complex of 47 proteins which is collectively known as the 26S proteasome (2.5 MDa). The 26S proteasome consists of a barrel-shaped 20S proteolytic core which is capped at one or both ends by 19S regulatory units (Voges et al., 1999). The β5, β2, and β1 protein subunits of the 20S core exhibit chymotrypsin-like, trypsin-like, and caspase-like activities, respectively (Figure 1A).

Disruption of proteasome function engenders toxic accumulation of damaged proteins and rapid build-up of incompatible regulatory proteins, which triggers the apoptotic cascade. Compared to normal cells, cancer cells are generally equipped with higher proteasomal capacity because they require upregulated protein quality control to survive under stress of growth and turnover. This phenomenon also renders them more sensitive to the pro-apoptotic effects of proteasome inhibition than normal cells-a strategy which has been successfully employed to treat multiple myeloma (MM) using proteasome inhibitors. Bortezomib (Velcade) is the first-in-class proteasome inhibitor which has contributed substantially to improved survival in MM patients in recent years (Moreau et al., 2012).

Many second generation proteasome inhibitors are under development with a goal to bring more potent, specific, and less toxic agents with diverse catalytic target within the 26S proteasome. Examples of such drugs include carfilzomib (PR-171) and ixazomib (MLN9708), which appear to exhibit low incidence of peripheral neuropathy, enhanced efficacy, ability to overcome bortezomib resistance (Lawasut et al., 2012). Diphenyldihaloketones CLEFMA and EF24 belong to a novel class of NF-κB inhibitors; a putative mechanism for their anti-NF-κB activity is the inhibition of the β isoform of inhibitor of κB kinase (IKKβ; Kasinski et al., 2008; Vilekar et al., 2012). CLEFMA and EF24 were developed as synthetic analogs of curcumin, a dietary component (Lagisetty et al., 2010; Olivera et al., 2012). Whereas proteasome-inhibitory activity of curcumin has been reported, similar effect of CLEFMA and EF24 has not been investigated. Intrinsic characteristics of drug-target interactions are such that a drug binds many targets, and conversely, a given protein target has a capacity to bind many drugs (Zhou et al., 2015). Close examination of the structural features of diphenyldihaloketones studied by our group (Lagisetty et al., 2010) revealed a potential target in proteasome. CLEFMA and EF24 possess α,β-unsaturated carbonyl functionality which imparts strong reactivity for nucleophilic attack and 1,4-additions in biological systems. On the other hand, the two central catalytic rings in the 20S core unit of proteasome exhibit nucleophilic character which is exploited by epoxomicin to selectively and irreversibly inhibit chymotrypsin-like proteasome activity (Groll et al., 2000). In this work, we started with a broad objective of investigating the proteasome-inhibitory activity of CLEFMA and EF24 and were successful in identifying regulatory subunit RPN13 as a putative target among 47 individual proteins comprising the 26S proteasome. Significant rationale to this work was provided by our concurrent observation that CLEFMA and EF24 prevent the 26S proteasome disassembly observed in gut tissue of rats with class IV hemorrhagic shock (Rao et al., 2018).

## Materials and methods

Synthesis and characterization of CLEFMA and EF24 have been described elsewhere (Lagisetty et al., 2009, 2010). Synthesis of biotin-analogs of CLEFMA and EF24 (Figure 1B) is described in supplemental material. We employed N-biotinylation of these compounds at a position away from their purported pharmacophore comprising of dienone moiety. Biotinylation enabled the use of streptavidin chemistry to monitor the compounds in various assays. The synthesized compounds were characterized by NMR and mass spectroscopy. Highly purified human 26, 20, and 19S proteasomes were obtained from Boston Biochem (Cambridge, MA). Proteasome subunit RPN13 containing pleckstrin-like receptor for ubiquitin (human RPN13-Pru) was acquired from MBL International (Woburn, MA). Specific fluorogenic peptide substrates for proteasome activity assays were purchased from Enzo Life Sciences (Farmingdale, NY). Antibody against rat and human RPN13 was obtained from Abcam (Cambridge, MA). Antibodies against ubiquitin and green fluorescent protein (GFP) were purchased from Santa Cruz Biotechnology (Santa Cruz, CA, USA). Anti-actin primary antibody, horse radish peroxidase (HRP)-conjugated anti-rabbit IgG secondary antibody, and HRP-streptavidin were from Sigma-Aldrich (St. Louis, MO). The immunoreactive bands were detected by SuperSignal West Femto detection reagent (Thermo Fischer Scientific, Rockford, IL). The immunoblots were imaged using Ultraquant image acquisition machine (Claremont, CA). Human umbilicus vessel endothelial cells (HUVEC), Ub^G76V^-GFP (GFPu-1) cells, human lung adenocarcinoma H441 cells, and rat small intestine epithelial cells (IEC-6 cells) were obtained from ATCC (Manassas, VA). Most other chemicals were sourced from various vendors represented by VWR (Radnor, PA). Animal experiments were conducted in accordance with NIH animal care and use guidelines and were approved by the Institutional Animal Care and Use Committee at the University of Oklahoma Health Sciences Center.

### Proteasome assay

Proteasome activity was measured by adapting a method described elsewhere (Kisselev and Goldberg, 2005). Substrates used for trypsin-like, chymotrypsin-like, and caspase-like enzyme activities were Boc-Leu-Arg-Arg-AMC, Suc-Leu-Leu-Val-Tyr-AMC, and Z-Leu-Leu-Glu-AMC, respectively. Approximately 100 ng of purified proteasome was incubated with 100 μL of assay buffer (50 mM Tris-HCl, pH 7.5, 40 mM KCl, 1 mM dithiothreitol, 0.5 mg/ml bovine serum albumin) in presence of 2 mM ATP and 5 mM MgCl_2_. The fluorogenic peptides were added at 1 mM working concentration in each well and the plate was incubated at 37°C. Fluorescence reading was monitored at 380–440 nm for up to 60 min. Boiled 26S proteasome (to kill enzyme activity) and bortezomib (a known proteasome inhibitor) were used as assay controls. To examine the effect of compounds on the activity of purified proteasomes, the 26S proteasome preparations were allowed to interact with CLEFMA or EF24 (10–50 μM, 15 min at room temperature) before the activity assay. The results were analyzed by one-way analysis of variance (ANOVA) applying the Bonferroni or Tukey *post-hoc* test using Prism 6 software (GraphPad, San Diego, CA, USA). A *p* < 0.05 was considered statistically significant.

### Treatment of GFPu-1 cells and HUVEC with CLEFMA and EF24 in culture

HUVEC and proteasome activity reporter GFPu-1 cells were grown to 70–80% confluence in a humidified atmosphere of 5% CO_2_ at 37°C. GFPu-1 cells were treated with 10 μM concentration of CLEFMA or EF24 for 0.5–4 h, whereas HUVEC cells were treated for 0.5–2 h. Cells were collected in RIPA (radioimmunoprecipitation assay) lysis buffer and separated on polyacrylamide gels for immunoblotting.

### Denaturing gel electrophoresis and immunoblotting

Immunoblotting of samples separated by denaturing gel electrophoresis was performed to examine the expression of ubiquitinated-proteins, RPN13, GFP, or streptavidin-reactive biotinylated chemicals. Expression of action was taken as a loading control for immunoblots. Briefly, the samples were collected in ice-cold RIPA buffer containing protease and phosphatase inhibitors (0.1 M phenylmethylsulfonyl fluoride, 0.2 M sodium orthovanadate, 1 M NaF, 2 μg/mL aprotinin, and 2 μg/mL leupeptin). After homogenization on ice, the protein was extracted by centrifugation at 18,000 × g for 15 min. The lysates were boiled in gel-loading dye (40% glycerol, 240 mM Tris/HCl, pH = 6.8, 8% SDS, 0.04% bromophenol blue, 5% β-mercaptoethanol), and fractionated on 10% polyacrylamide gels. The separated proteins were electro-transferred on to nitrocellulose membranes. The membranes were blocked with 5% fat-free milk for 1 h and probed overnight with primary antibodies, followed by 1 h probing with secondary antibody.

### Interaction of CLEFMA and EF24 with proteasome

Biotin-tagged CLEFMA or EF24 (10 μM) were allowed to interact with 100 ng of purified human proteasome preparations for 10–30 min at room temperature. Vehicle (dimethylsulfoxide) used for dissolving compounds was used in control interaction reactions. For drug interaction in whole cell lysate matrix, IEC-6 cells were cultured according to the supplier's recommendations and lysed in RIPA buffer. Approximately 100 μg of IEC-6 lysate protein was reacted with biotin-CLEFMA. Drug-proteasome and drug-lysate mixtures were separated by reducing denaturing gel electrophoresis, transferred onto nitrocellulose membranes, and probed with streptavidin-HRP or immunoblotted for RPN13 protein.

For pull down assays, biotin-CLEFMA or -EF24 were allowed to react with human 26S proteasome or IEC-6 cell lysate as described above and the mixtures were incubated overnight with streptavidin-sepharose beads. The complex captured by the beads was eluted in 0.1 M glycine buffer and electrophoresed for staining with HRP-streptavidin or blotting with human- or rat-reactive RPN13 antibody.

### Two dimensional (2D) polyacrylamide gel electrophoresis (PAGE)

Purified human 19S proteasome was allowed to react with biotin-CLEFMA and the reaction mixture was submitted to Kendrick labs (Madison, WI). Two-dimensional electrophoresis was performed according to the carrier ampholine method of isoelectric focusing (O'Farrell, 1975; Burgess-Cassler et al., 1989) by Kendrick Labs, Inc. (Madison, WI) as follows: Isoelectric focusing (IEF) was carried out in a glass tube of inner diameter 2.0 mm using 2% pH 3-10 ampholines mix (GE Healtcare, Piscataway, NJ and Serva, Heidelberg, Germany) for 9,600 volt-h. One μg of an IEF internal standard, tropomyosin, was added to the sample. This protein migrates as a doublet with lower polypeptide spot of MW 33,000 and pI 5.2. The enclosed tube gel pH gradient plot for this set of ampholines was determined with a surface pH electrode.

After equilibration for 10 min in buffer ‘O’ (10% glycerol, 2.3% SDS and 0.0625 M tris, pH 6.8), each tube gel was sealed to the top of a stacking gel that overlaid a 10% acrylamide slab gel (0.75 mm thick). SDS slab gel electrophoresis was carried out in duplicate for about 4 h at 15 mA/gel. One gel was used for Coomassie staining and the other gel was used for blotting. Myosin (220,000), phosphorylase A (94,000), catalase (60,000), actin (43,000), carbonic anhydrase (29,000), and lysozyme (14,000) were used as molecular weight standards. These standards appear along the basic edge of the Coomassie blue-stained 10% acrylamide slab gel. The stained gel was dried between sheets of cellophane with the acid edge to the left. The duplicate gel for blotting was placed in transfer buffer (10 mM Caps, pH 11.0, 10% MeOH) and transferred onto a PVDF membrane overnight at 200 mA and ~100 volts/two gels. Like in the first gel, the molecular weight standards appear as bands at the basic edge of the Coomassie Brilliant Blue R-250-stained membrane.

The Coomassie-stained membrane was scanned, de-stained in 100% methanol, and rinsed briefly in Tween 20 tris-buffered saline (TTBS). The membrane was blocked for 2 h in 5% Bovine Serum Albumin (BSA) in TTBS, before 2 h incubation in primary solution of poly-HRP Streptavidin [ThermoFisher Sci., Waltham, MA diluted 1:500,000 in 2% BSA in TTBS]. The membrane was rinsed three times for 10 min each in TTBS, treated with enhanced chemilumescent reagent, and exposed to x-ray film.

### Protein digestion and peptide extraction

Proteins that were separated by 2D-PAGE and stained by Coomassie dye were excised, washed and the proteins from the gel were treated according to published protocols (Shevchenko et al., 1996; Darie et al., 2011; Sokolowska et al., 2012c). Briefly, the gel pieces were washed in high purity HPLC grade water, dehydrated, cut into small pieces, and destained by incubating in 50 mM ammonium bicarbonate, 50 mM ammonium bicarbonate/50% acetonitrile, and 100% acetonitrile under moderate shaking, followed by drying in a speed-vac concentrator. The gel bands were rehydrated with 50 mM ammonium bicarbonate. The procedure was repeated twice. The gel bands were then rehydrated in 50 mM ammonium bicarbonate containing 10 mM DTT and incubated at 56°C for 45 min. The DTT solution was then replaced by 50 mM ammonium bicarbonate containing 100 mM iodoacetamide for 45 min in the dark, with occasional vortexing. The gel pieces were then re-incubated in 50 mM ammonium bicarbonate/50% acetonitrile, and 100% acetonitrile with moderate shaking, followed by drying in speed-vac concentrator. The dried gel pieces were rehydrated using 50 mM ammonium bicarbonate containing 10 ng/μL trypsin and incubated overnight at 37°C under low shaking. The resulting peptides were extracted twice with 5% formic acid/50 mM ammonium bicarbonate/50% acetonitrile and once with 100% acetonitrile under moderate shaking. The peptide mixture was dried in a speed-vac and solubilized in 20 μL of 0.1% formic acid/2% acetonitrile.

### Liquid chromatography coupled with tandem mass spectroscopy (LC-MS/MS)

The entire procedure has been previously described (Darie et al., 2011; Sokolowska et al., 2012a,b). The peptide mixture was analyzed by reversed phase LC-MS/MS using a NanoAcuity UPLC system (Micromass/Waters, Milford, MA) coupled to a Quadrupole-Time of flight Ultima API MS (Micromass/Waters, Milford, MA). Briefly, the peptides mixture was loaded onto a 100 μm × 10 mm NanoAquity BEH130 C18 1.7 μm UPLC column (Waters, Milford, MA) and eluted over a 150 min gradient of 2–80% organic solvent (acetonitrile containing 0.1% trifluoroacetic acid) at a flow rate of 400 nL/min. The aqueous solvent was 0.1% trifluoroacetic acid in HPLC water. The column was coupled to a Picotip Emitter Silicatip nano-electrospray needle (New Objective, Woburn, MA). MS data acquisition involved survey MS scans and automatic data dependent analysis (DDA) of the top three ions with the highest intensity ions with the charge of 2+, 3+, or 4+ ions. The MS/MS was triggered when the MS signal intensity exceeded 10 counts/s. In survey MS scans, the three most intense peaks were selected for collision-induced dissociation (CID) and fragmented until the total MS/MS ion counts reached 10,000 or for up to 6 s each. Calibration was performed for both precursor and product ions using 1 pmol GluFib (Glu1-Fibrinopeptide B) standard peptide with the sequence EGVNDNEEGFFSAR and the monoisotopic doubly-charged peak with m/z of 785.84.

### MS data processing and protein identification

Raw data were processed using ProteinLynx Global Server (PLGS, version 2.4) software as previously described (Sokolowska et al., 2012a). The following parameters were used: background subtraction of polynomial order 5 adaptive with a threshold of 30%, two smoothings with a window of three channels in Savitzky-Golay mode and centroid calculation of top 80% of peaks based on a minimum peak width of 4 channels at half height. The resulting pkl files were submitted for database search and protein identification to the public Mascot database search (www.matrixscience.com, Matrix Science, London, UK) using the following parameters: databases from NCBI (bacteria), parent mass error of 1.3 Da, product ion error of 0.8 Da, enzyme used: trypsin, one missed cleavage, propionamide as cysteine fixed modification and Methionine oxidized as variable modification. To identify the false negative results, we used additional parameters such as different databases or organisms, a narrower error window for the parent mass error (1.2 and then 0.2 Da) and for the product ion error (0.6 Da), and up to two missed cleavage sites for trypsin. In addition, the pkl files were also searched against in-house PLGS database version 2.4 (www.waters.com) using searching parameters similar to the ones used for Mascot search. The Mascot and PLGS database search provided a list of proteins for each gel band. To eliminate false positive results, for the proteins identified by either one peptide or a mascot score lower than 25, we verified the MS/MS spectra that led to identification of a protein.

### Drug affinity responsive target stability (DARTS)

We employed DARTS technique to examine if CLEFMA binding to RPN13 confers protection against proteolysis catalyzed by thermolysin. DART is applicable to covalent interaction of test compound and its target protein. We hypothesized covalent interaction between CLEFMA/EF24 and RPN13 based on a recently reported similar interaction (Anchoori et al., 2013). Whole cell lysate of human lung adenocarcinoma H441 cells provided the matrix containing RPN13. Briefly, H441 cells cultured at 37°C with 5% CO_2_ in RPMI 1640 medium (Invitrogen, Carlsbad, California) supplemented with 10% heat-inactivated fetal bovine serum and gentamicin at 50 μg/ml. The cells were lysed with homogenization buffer supplemented with protease and phosphatase inhibitor. After centrifugation (13,500 rpm, 15 min), the lysate was diluted to the protein concentration of 5 mg/ml. The lysates was incubated with or without CLEFMA-biotin (100 μM) for 30 min, followed by addition of thermolysin (1 μg thermolysin for every 15 μg lysate) in reaction buffer [50 mM Tris·HCl, 50 mM NaCl, 10 mM CaCl_2_]. The samples were incubated at 37°C, and after indicated times, the reaction was stopped by addition of 6 × gel-loading dye and boiling the samples for 10 min. The samples were loaded on polyacrylamide gels for SDS-PAGE, for Coomassie Brilliant Blue staining or blotting with HRP-Streptavidin for chemiluminescence detection on a nitrocellulose membrane.

## Results

### CLEFMA and EF24 inhibit proteolytic activity of highly purified 26S proteasome

**Figure 2:**
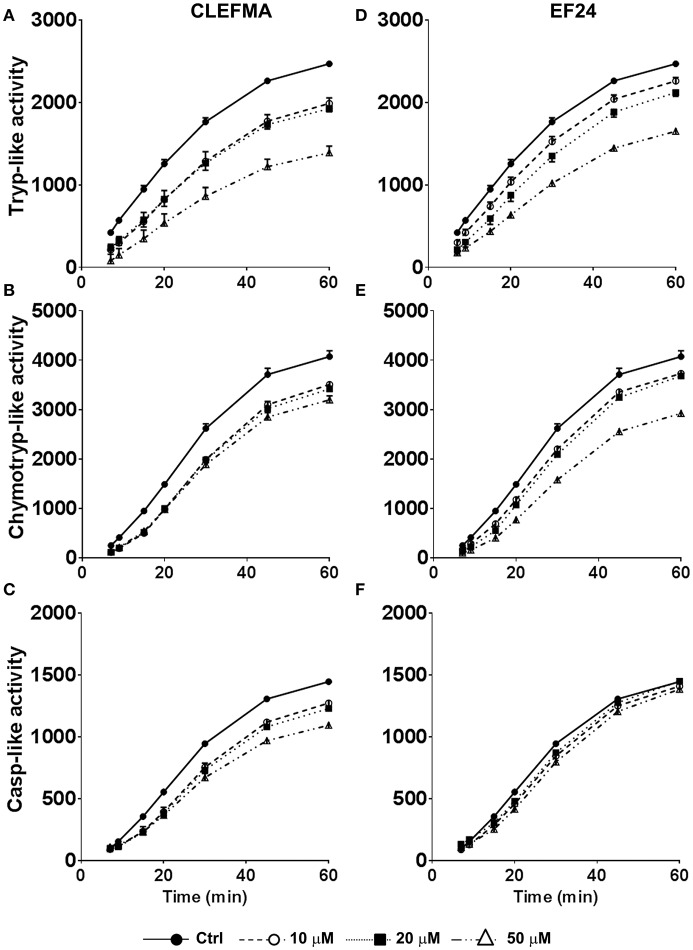
Effects of CLEFMA **(A,B,C)** and EF24 (**D,E,F**) on the 26S proteasome activity. The 26S proteasome was allowed to interact with CLEFMA or EF24 for 30 min; trypsin-like, chymotrypsin-like, and caspase-like activities were determined. Fluorescence emission was recorded up to 60 min of reaction.

We measured the trypsin-like, chymotrypsin-like, and caspase-like proteolytic activities of purified 26S proteasome after allowing it to interact with CLEFMA or EF24 (0, 10, 20, and 50 μM) at room temperature. As shown in Figure 2, treatment with CLEFMA and EF24 significantly altered the activity profile for trypsin- and chymotrypsin-like activities in a dose-dependent manner. As compared to untreated 26S proteasome, drug treatment significantly reduced trypsin- and chymotrypsin-like activities (*p* < 0.001). Although caspase-like activity was not altered by EF24 treatment (*p* = 0.9), CLEFMA was effective in significantly reducing caspase-like activity also (*p* < 0.001). Further analysis of proteasome-inhibition revealed that CLEFMA inhibits trypsin- and chymotrypsin-like activities with IC50 values of 60.7 μM and 124.6 μM, respectively. Corresponding IC50 values for EF24 were 415.7 μM and 127.0 μM, respectively (Figure S1).

### CLEFMA and EF24 inhibit 26s proteasome in GFPu-1 and HUVEC cells

**Figure 3:**
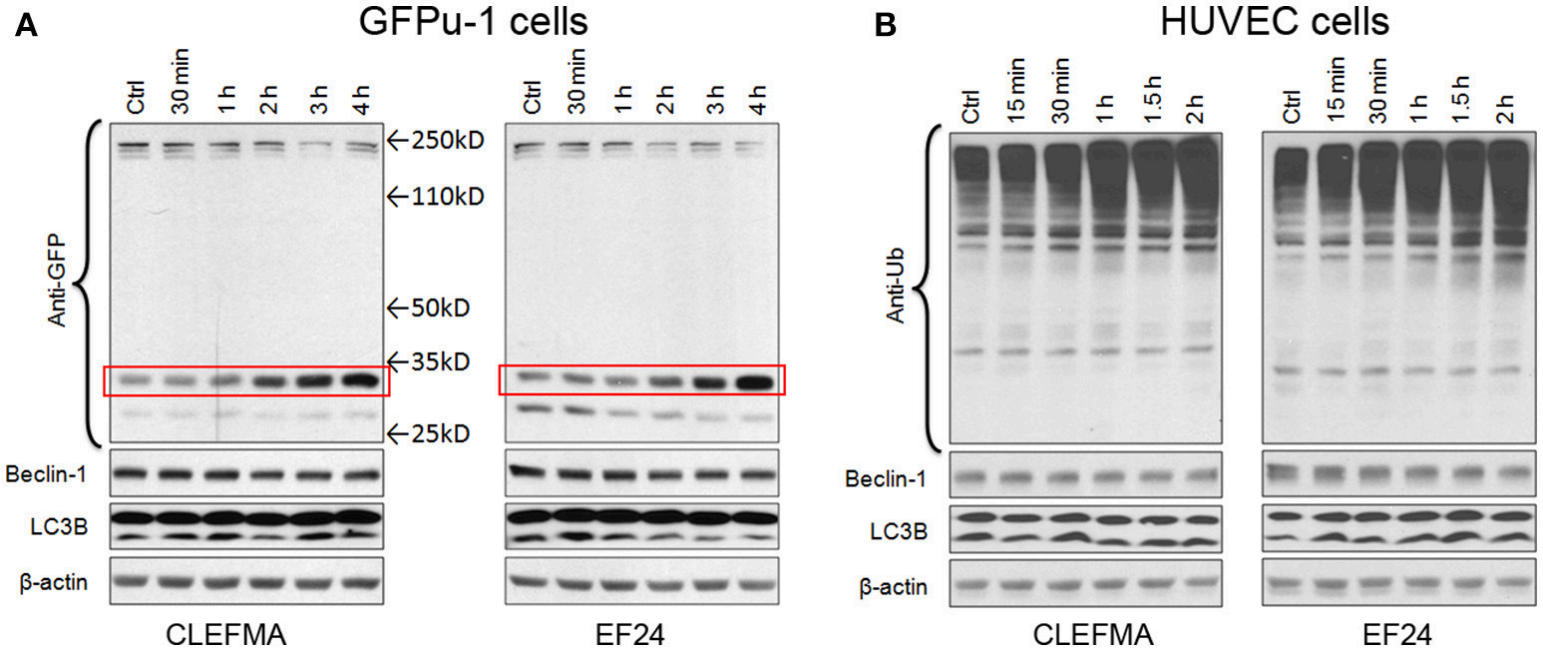
CLEFMA and EF24 inhibit proteasome *in vivo*. **(A)** GFPu-1 and **(B)** HUVEC cells were treated with CLEFMA or EF24 (10 μM) and the cell lysate was probed by immunoblotting with anti-ubiquitin antibody. The protection of GFP over time from proteasome-mediated degradation is highlighted by a surrounding box.

We examined the effect of CLEFMA and EF24 in standard proteasome reporter cell lines GFPu-1 and HUVEC. GFPu-1 cells express ubiquitinated green fluorescent protein (Ub-GFP) which is constitutively degraded by endogenous proteasome. When GFPu-1 cells were treated with 10 μM of CLEFMA or EF24, the stability of Ub-GFP increased and it accumulated in cells over time (Figure 3A). Given the molecular weights of a single Ub (8.5kD) and GFP (27kD), the bands shown in Figure 3A represent mono-ubiquitinated GFP. In HUVEC cells also, CLEFMA and EF24 treatments increased the accumulation of endogenously expressed ubiquitinated proteins in HUVEC cell line (Figure 3B). These *in vivo* results confirmed that diphenyl dihaloketones interfere with proteasome activity.

Autophagy (or macroautophagy) is a catabolic process where the cytoplasmic contents are sequestered within autophagolysosomes and degraded in the lysosomes (Debnath et al., 2005). Expression of ATG6 orthologue beclin is regarded as a marker for autophagy. Similarly, an increase in lower LC3B-II with respect to upper LC3B-I band is a sign of autophagy induction. In GFPu-1 and HUVEC cells, we found no alteration in the expression of beclin-1 and electrophoretic mobility of LC3B.

### CLEFMA and EF24 interact with 19S regulatory unit

**Figure 4:**
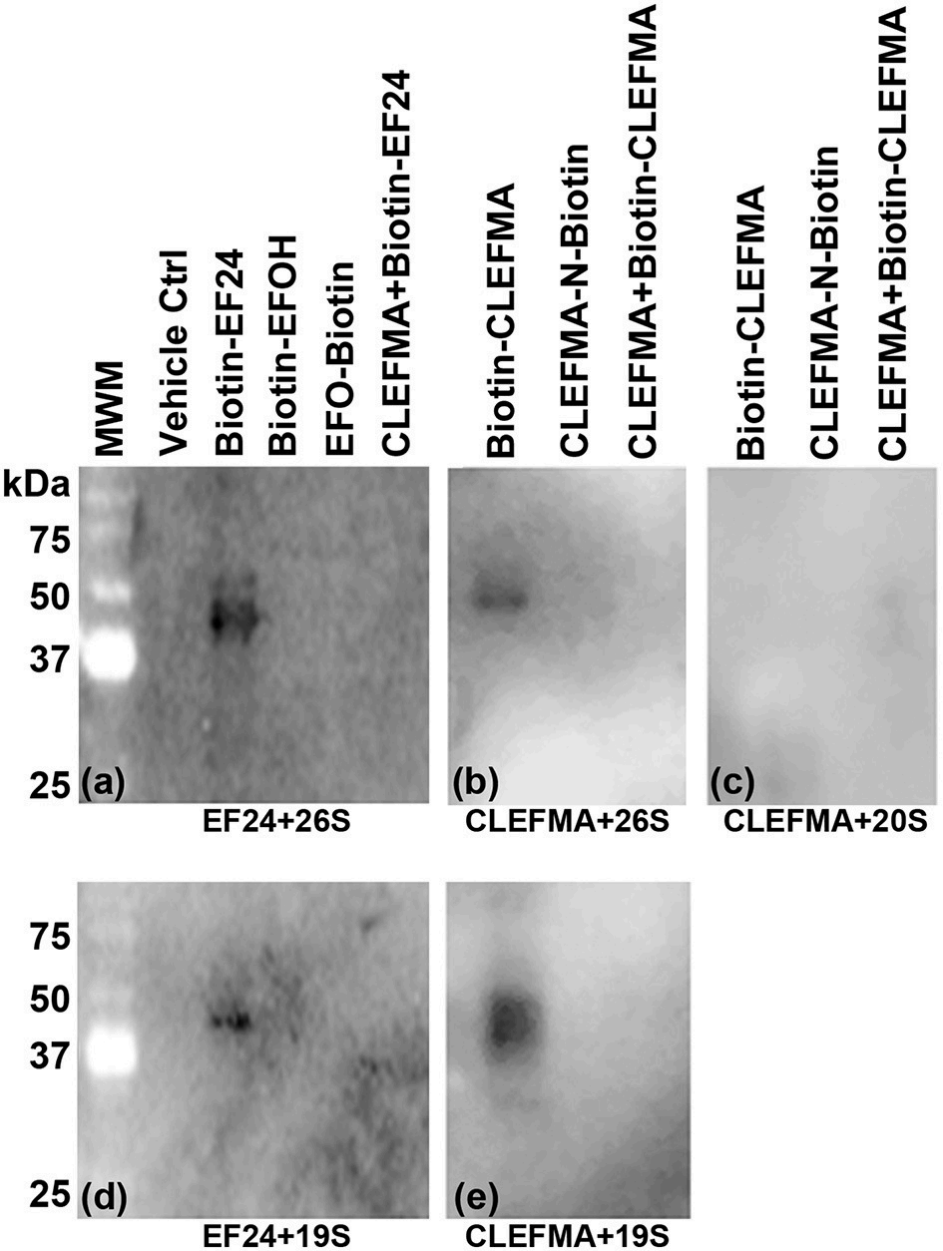
Interaction of EF24 and CLEFMA with proteasome. Highly purified **(A,B)** 26S proteasome, **(C)** 20S proteasome, and **(D,E)** 19S regulator were allowed to interact with biotinylated analogs of EF24 or CLEFMA (10 μM). The mixtures were separated on a regular 10% reducing gel and the transfer membranes were probed for biotin using HRP-streptavidin.

To investigate the drug-target interaction, we employed several traceable CLEFMA and EF24 probes labeled with biotin. The chemical structure and definition of these drug analogs is given in Figure 1B. We allowed biotin-CLEFMA and biotin-EF24 to interact with the highly-purified preparations of human 26 and 20S proteasome. The reaction mixtures were separated on a gel and the separated proteins were probed by streptavidin-HRP. As shown in Figure 4, we found that the biotinylated compounds interacted with a 43 kDa protein in the 26S proteasome (lane 3, Figure 4A and lane 1 of Figure 4B). EFOH, a known inactive analog of EF24, did not interact with the 26S proteasome (lane 4, Figure 4A). Moreover, CLEFMA or EF24 biotinylated at the pharmacophore (EFO-Biotin and CLEFMA-N-Biotin) did not show any interaction with the 26S proteasome (lane 5, Figure 4A and lane 2, Figure 4B). At the same time, presence of a 10-fold excess of untagged CLEFMA displaced biotin-EF24 and biotin-CLEFMA from the drug-proteasome complexes (lane 6, Figure 4A and lane 3, Figure 4B). However, there was no interaction of these compounds with 20S proteasome; only CLEFMA+20S interaction is shown (lane 1, Figure 4C).

**Figure 5:**
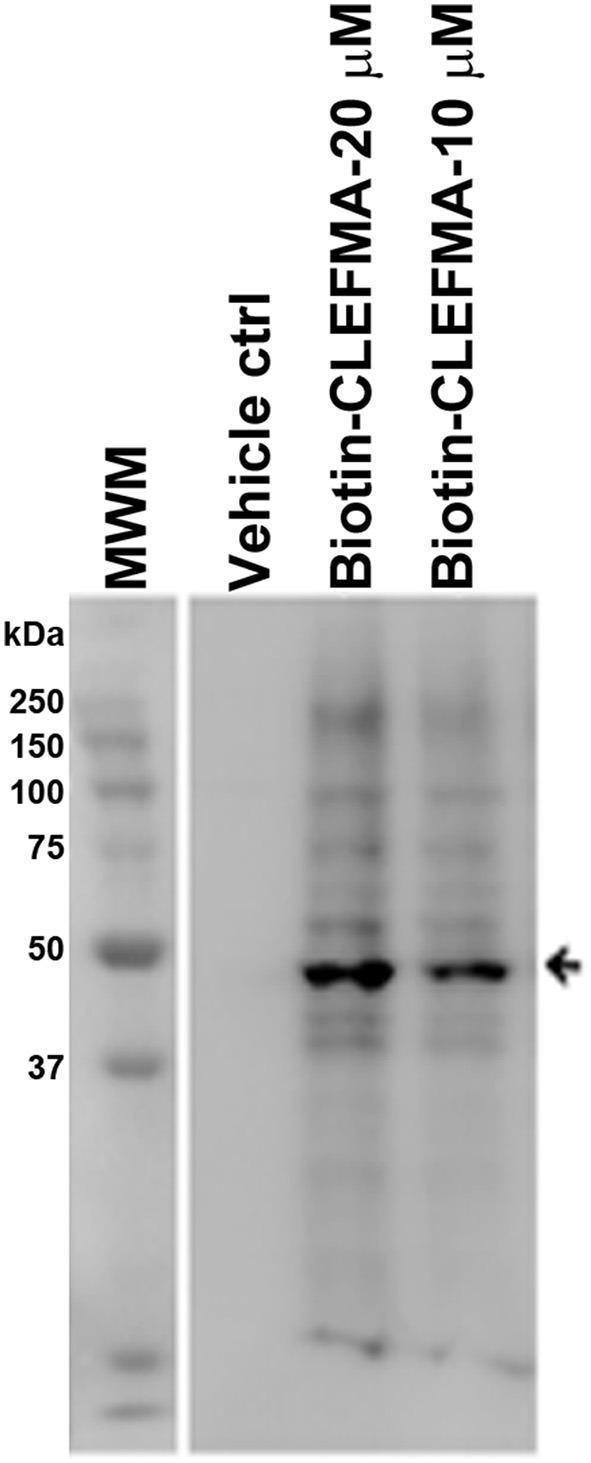
IEC-6 cell lysate was incubated with biotin-CLEFMA (10 and 20 μM) or CLEFMA-N-biotin (10 μM) and separated on a gel. After transferring to a membrane, the proteins were probed HRP-streptavidin.

The above observations and the fact that the only difference between the 26S and the 20S proteasome is the presence of 19S cap led us to hypothesize that the target protein for CLEFMA and EF24 perhaps exists in the 19S unit. To test this hypothesis we allowed CLEFMA and EF24 to interact with purified 19S proteasome. As shown in Figures 4D,E, both EF24 and CLEFMA were found to show interaction identical to that shown with the 26S proteasome, i.e., the two compounds interacted with a 43 kDa protein in the 19S proteasome. In order to confirm a 43 kDa interacting partner of these compounds, we further examined this interaction in IEC-6 cells by incubating biotin-CLEFMA (10 and 20 μM) with whole cell lysate (Figure 5). We found that biotin-CLEFMA prominently reacted with a 43 kDa protein in the cell preparation, whereas inactive biotinylated analog of CLEFMA did not show any interaction. The other weak intensity bands could be the non-specific interaction of streptavidin-HRP probe with endogenous biotinylated proteins (Weissman, 2001).

### RPN13 is the putative 43kDa target of CLEFMA in the 19S regulatory unit

**Figure 6:**
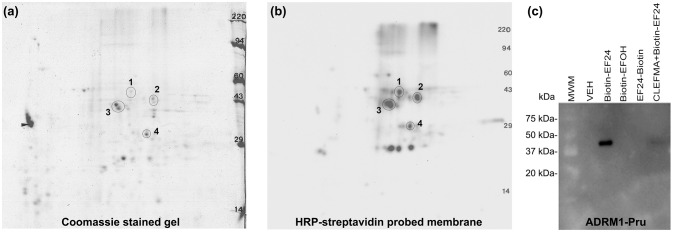
CLEFMA and EF24 interact with RPN13. Purified 19S regulatory particle was allowed to react with biotin-CLEFMA and the reaction mixture was separated by 2D-gel electrophoresis. **(a)** Coomassie-stained 2D-gel and **(b)** Corresponding transfer membrane probed with HRP-streptavidin. The four spots identified in the pictures were subjected to mass spectroscopic analysis (Table S1). **(c)** Recombinant human RPN13-Pru protein was reacted with various biotin-EF24 in presence (lane 6) or absence (lane 3) of free CLEFMA. Inactive biotinylated analogs of EF24 were also reacted as controls.

Having realized that the target protein for interaction with CLEFMA and EF24 resides in the 19S unit, we subjected the reaction mixture of the 19S protein and biotin-CLEFMA to 2D-gel electrophoresis. Coomassie-stained and HRP-streptavidin probed 2D-gels are shown in Figures 6a,b respectively. Among the four major spots observed on the gel, a prominent spot #3 was close to 43 kDa. Mass spectroscopic analyses identified spot #3 as RPN13, a non-ATPase regulatory subunit of 19S of molecular weight 43,246 Da. The mascot score for RPN13 identity was 923 and the number of peptide sequences identified were 26 (Table S1). To confirm this interaction between diphenyldihaloketones and RPN13, we incubated biotin-EF24 with recombinant human RPN13-Pru protein, separated on gel and performed blotting with HRP-streptavidin. As shown in Figure 6c, RPN-Pru conspicuously interacted with EF24 and the band corresponded to the molecular weight (42 kDa) of RPN-Pru (lane 3); inactive biotinylated analogs of EF24 were not found to interact with RPN13-Pru (lanes 4 and 5) and presence of free CLEFMA was found to displace biotin-EF24 from binding with RPN13-Pru (lane 6).

**Figure 7:**
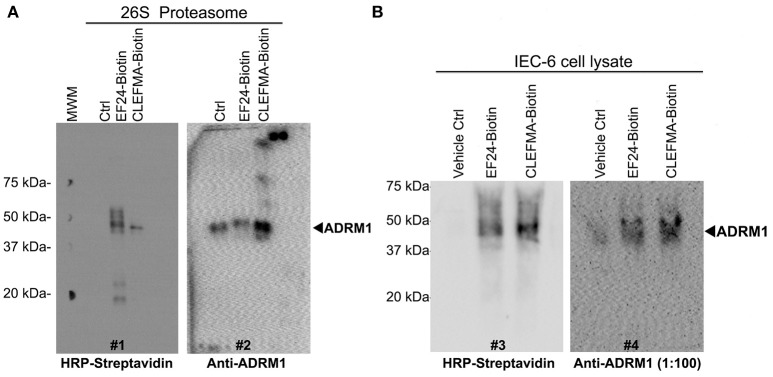
CLEFMA and EF24 interact with RPN13 in the fully assembled 26S proteasome and whole cell lysate. **(A)** The biotinylated compounds were allowed to react with purified human 26S proteasome, the mixture was separated on poly-acrylamide gels, and the transfer membrane was probed with HRP-streptavidin (blot #1). The blot was stripped and re-probed with anti-human RPN13 (blot #2). **(B)** Streptavidin bead-mediated pull down of RPN13 from rat IEC-6 cell lysate treated with biotinylated EF24 or CLEFMA. After blotting with anti-human HRP-streptavidin (blot #3), the membrane was stripped and re-probed with anti-rat RPN13 antibody (blot #4).

To further scrutinize RPN13 as the target of diphenyldihaloketones, we performed a co-localization experiment. Human 26S was allowed to interact with biotin-CLEFMA or biotin-EF24 and the drug-protein complex was separated on a non-reducing native 10% gel, with an expectation that RPN13 will be co-localized on the membrane by both HRP-streptavidin and anti-RPN13 antibody. As shown in Figure 7A, a streptavidin-reactive band at ~43 kDa was visible in lanes containing biotinylated compounds (blot #1), and a band of same size also appeared upon re-blotting the membrane with anti-RPN13 antibody (blot #2). As expected, the control preparation without any drug only stained with anti-RPN13 antibody to show the correct band of RPN13 protein. We validated these results by a pull-down study using whole cell lysate (Figure 7B). IEC-6 cell lysate was allowed to react with CLEFMA-biotin or EF24-biotin, the mixture was pulled down by using streptavidin beads, and the captured complex was probed for the presence of biotin and RPN13 by probing with HRP-streptavidin and anti-rat RPN13 antibody. Clearly, the results showed co-localization of biotin signal with that of RPN13 protein (Figure 7B). Together these results provided strong evidence to propose RPN13 protein of 19S unit of proteasome as a putative target for diphenyldihaloketone compounds.

### Interaction with CLEFMA does not protect RPN13 from thermolysin-mediated proteolysis

**Figure 8:**
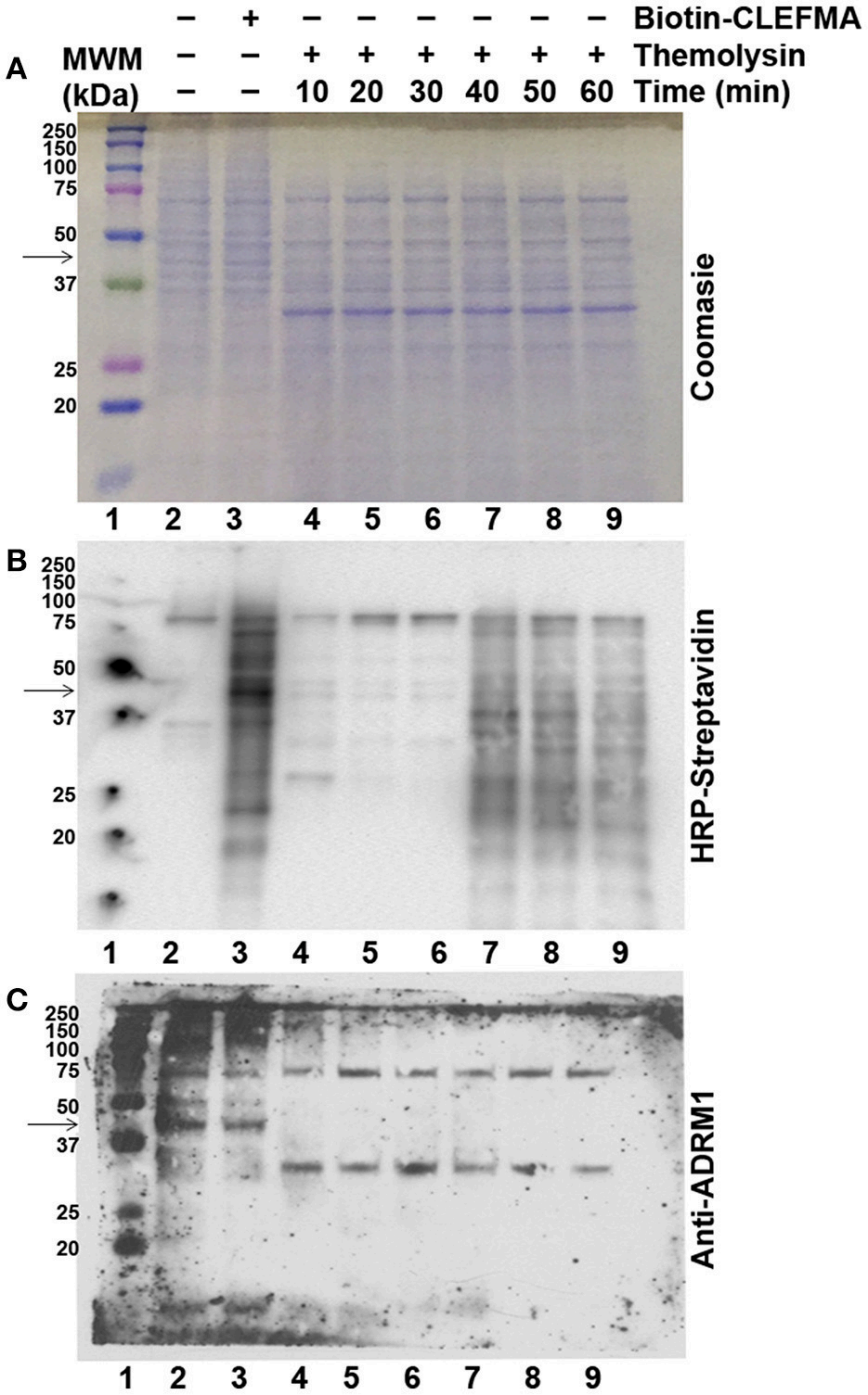
Effect of CLEFMA on proteolytic stability of RPN13. Whole cell lysate of H441 cells was treated with biotin-CLEFMA, followed by thermolysin digestion at room temperature for 10–60 min. The mixture was separated on an acrylamide gel, and the separated proteins were subjected to **(A)** Coomassie Brilliant staining, **(B)** HRP-Streptavidin probing for chemiluminescence imaging, and **(C)** Anti-RPN13 immunoblotting.

In order to investigate whether CLEFMA binding protects RPN13 protein against proteolytic enzymes, we allowed H441 cell lysate to interact with biotin-CLEFMA and subjected the lysate to thermolysin digestion. Proteolytic activity of thermolysin was evident by loss of high molecular weight bands in lanes 4–9 compared to lanes 2–3 of the Coomassie-stained gel (Figure 8A). However, there was no evidence of any specific protein being preserved after treatment with biotin-CLEFMA. Appearance of a new protein band at ~30 kDa is characteristic of thermolysin. When the transferred proteins were probed with HRP-streptavidin on a membrane, we observed biotin-CLEFMA interacting with 43 kDa protein (RPN13), but thermolysin treatment obliterated this band (lane 3 vs. lanes 4–9, Figure 8B). When the same membrane was immunoblotted with anti-RPN13 antibody, there was a clear presence of the 43 kDa target protein in samples without thermolysin treatment, but this protein disappeared in samples treated with thermolysin (lanes 2–3 vs. lanes 4–9, Figure 8C).

## Discussion

Fully assembled holo-complex of eukaryotic proteasome can exist as 26S which is capped on one end or 30S which is capped on both ends with 19S regulatory particle (Figure 1). Most known proteasome inhibitors, such as bortezomib and epoxomicin, interact with β5 catalytic protein in the 20S core and inhibit chymotrypsin-like activity. Inhibition of trypsin-like activity and caspase-like activity is minimal at pharmacologic doses of these compounds, but occurs at higher concentrations and slower rate (Lawasut et al., 2012). Results of this study demonstrate that CLEFMA and EF24 moderately inhibit trypsin as well as chymotrypsin-like activity of the 26S proteasome.

Structural features of proteasome inhibitors of peptide aldehyde, vinyl sulfone, boronate, glyoxal, and α′,β′-epoxyketone classes relative to catalytic sites of the proteasome have been discussed in several places (Meng et al., 1999; Kisselev et al., 2012). At very basic level, these compounds mimic natural substrates of catalytic β-subunits. In contrast, small molecules CLEFMA and EF24 are not peptides and are not expected to mimic as proteolytic substrates. A previous structure activity relationship study identified dienone moiety of CLEFMA and EF24 as their pharmacophore (Lagisetty et al., 2010). Here also, we found that compromising this dienone pharmacophore in CLEFMA and EF24 resulted in analogs that were unable to interact with proteasome in direct binding assays. Interestingly, CLEFMA and EF24 primarily inhibited trypsin-like activity (Figure 2). Known inhibitors of trypsin-like activity are typically peptides containing basic amino acids for interference with β2 subunit of proteasome (Mirabella et al., 2011). A peptidomimetic maleoyl-β-alanyl-valyl-arginal is an example of a selective inhibitor of trypsin-like activity of proteasome (Loidl et al., 1999). However, it is unusual for a small molecule to directly influence trypsin-like sites of proteasome because there is no known mechanism to translocate them into the 20S core. These unique observations about CLEFMA and EF24 led us to hypothesize that the two influence proteasome activity without directly interacting with β-subunits. Direct binding assays between biotinylated compounds and purified proteasome preparations provided a strong evidence for the presence of a molecular interaction of CLEFMA and EF24 with the 19S regulatory particle of the 26S proteasome.

The 19S regulatory particle, also known as PA700, is composed of 19 protein subunits which are grouped into RPN class (RPN1-13) and RPT class (RPT1-6). Although function of each individual subunit is not completely understood, collectively they regulate access of substrates to proteolytic active sites in the 20S core. A protein becomes a proteasome substrate after polyubiquitination with Lys48-linked ubiquitin. The ubiquitinated protein docks onto the 19S unit via ubiquitin receptors populating the 19S cap. RPN10 and RPN13 subunits are two ubiquitin receptors for substrates that are Lys48-linked ubiquitinated (Sakata et al., 2012). After binding to either of these receptors, other associated enzymes cause deubiquitination and linearization of the docked protein in an ATP-dependent process. The six subunits of RPT class exhibit ATPase activity; they help to gate the channel and translocate substrates into the catalytic 20S core (Bar-Nun and Glickman, 2012). We identified a 43 kDa molecular target in the 19S particle which interacts with CLEFMA and EF24 to inhibit proteolytic activity of the proteasome by a unique mechanism. Among various 19S subunits, (Table S2), the molecular masses of four non-ATPase regulatory subunits (RPN10, RPN7, RPN9, and RPN13) and two ATPase regulatory subunits (RPT6 and RPT4) fall reasonably close to 43 kDa. Evidence from LC-MS/MS, pull down assays, and further inquiry via direct binding assays identified RPN13 as the most plausible target for CLEFMA and EF24. Covalent attachment of small molecule inhibitors to their protein binding site is known to protect the protein against proteolysis by enzymes. The technique of DARTS relies on this principle (Pai et al., 2015). A compound similar CLEFMA and EF24 is RA-190 which has been recently reported to bind to RPN13 in covalent fashion (Anchoori et al., 2013). Our results indicated that CLEFMA binding to its putative target RPN13 does not protect latter from proteolytic activity of thermolysin. Further work with biophysical techniques, such as isothermal calorimetry and nuclear magnetic resonance, will improve our understanding about the covalent nature of RPN13' interaction with CLEFMA and EF24.

Evidence is now emerging that RPN13 could be a viable target for anti-cancer drug development. RPN13 was first recognized as adhesion regulating molecule 1 or ADRM1 almost exclusively in soluble 26S proteasome of HeLa cells (Jorgensen et al., 2006). Later it was found to bind ubiquitin as well as ubiquitin-like (UBL) domains and serve as a receptor for deubiquitinating enzyme Uch37 (Husnjak et al., 2008). RPN13 is over-expressed in a variety of cancers, including MM, ovarian, cervical, pancreatic and colorectal cancer (Pilarsky et al., 2004; Song et al., 2016; Jiang et al., 2017). Recently, a peptoid called KDT-11 has been discovered as selective and reversible ligand for RPN13 with modest affinity (Trader et al., 2015). It displays synergism with bortezomib (Trader et al., 2015). Earlier, Anchoori et al identified an RPN13-binding compound called RA-190 which was found to be selectively and irreversibly toxic to MM cells (Anchoori et al., 2013). Although RA-190 is a tetrachlorobenzylidene analog very similar to CLEFMA and EF24, we found a significant difference in their effects on autophagy. RA-190 has been found to trigger autophagy in MM cells (Song et al., 2016). Conventional proteasome inhibitors targeting 20S core particle also invariably induce autophagy (Ge et al., 2009; Zhu et al., 2010; Hui et al., 2012; Bao et al., 2016). CLEFMA and EF24, on the other hand, had no apparent effect on the expression of autophagy markers LC3B and beclin in proteasome reporter cell lines (Figure 3). That this difference is because of the difference in cells used in the two studies is a distinct possibility. Nevertheless the observation is significant since induction of compensatory autophagy by proteasome inhibitors has been regarded as one reason for emergence of bortezomib (Zang et al., 2015; Frassanito et al., 2016). Accordingly, autophagy may act as a backup system for the UPS (Lilienbaum, 2013).

In conclusion, CLEFMA and EF24 represent a unique set of proteasome inhibitors which influence the 19S regulatory particle of the 26S proteasome. Biological consequences and therapeutic implications of proteasome inhibition are far and wide, especially in oncology (Lawasut et al., 2012; Moreau et al., 2012). By virtue of the proteasomal hold on various mediators in the NF-κB pathway, proteasome inhibitors have anti-inflammatory activity. Proteasome function determines the stability of IκBα, processing of p105/100 NF-κB units, and degradation of nuclear p65 NF-κB protein (Saccani et al., 2004; Lawrence, 2009; Sun, 2017). Interestingly, EF24 was originally implicated to interact with IKKβ resulting in inhibition of NF-κB pathway (Kasinski et al., 2008). In light of the evidence provided in this article, the mechanism of NF-κB inhibition by CLEFMA and EF24 may be partly through their action on proteasome.

An under-researched application of proteasome inhibitors is in hemorrhagic shock. Mechanistically different, CLEFMA, and EF24 have shown efficacy in overcoming systemic inflammatory response and multi-organ dysfunction in a rat model of hemorrhagic shock (Yadav et al., 2013, 2014). It is notable that CLEFMA and EF24 treatment restored the hemorrhagic shock-induced proteasome activity in gut epithelium (Rao et al., 2018). At the same time, identification of RPN13 as the drug target brings to fore a few concerns related to the extra-proteasomal functions of RPN13 (Lamerant and Kieda, 2005; Kim et al., 2009). As noted elsewhere, RPN13 was originally identified as an adhesion-regulating molecule. In this capacity, its expression induces lymphocyte-endothelial interaction (Lamerant and Kieda, 2005). Study of such extra-proteasomal phenomena will be particularly relevant as such RPN13-targeting compounds develop into drugs.

## Author contributions

GN and VA synthesized biotinylated probes and prepared samples for 2D gel electrophoresis and LC-MS/MS. GR, VA, HY, and CT performed interactions studies. JX conducted proteasome assays in cells. HY, HH, CT, GR, and VA performed cell-free proteasome assays. VA, GR, and GN wrote the manuscript.

### Conflict of interest statement

The authors declare that the research was conducted in the absence of any commercial or financial relationships that could be construed as a potential conflict of interest.
