# Supplementary material for: Ubiquitin Receptor RPN13 Mediates the Inhibitory Interaction of Diphenyldihaloketones CLEFMA and EF24 With the 26S Proteasome
Source: Front Chem. 2018 Sep 10;6:392. doi: 10.3389/fchem.2018.00392 (PMC6153970; doi:10.3389/fchem.2018.00392)
Supplement: Supplementary file 1 [file Data_Sheet_1.DOCX]

***Supplemental Material***

**Ubiquitin Receptor RPN13 Mediates the Inhibitory Interaction of Diphenyldihaloketones CLEFMA and EF24 with the 26S Proteasome**

Geeta Rao, Gregory Nkepang, Jian Xu, Hooman Yari, Hailey Houson, Chengwen Teng, and Vibhudutta Awasthi^*^

*** Correspondence:** Vibhudutta Awasthi: vawasthi@ouhsc.edu

**1 Synthesis of biotinylated analogs of CLEFMA and EF24**

**1.1 CLEFMA-PEG_2_-Biotin (1)**

To 2 mL of anhydrous dimethylformamide (DMF) were added CLEFMA (29.46 mg, 0.067 mmol), biotin-PEG_2_-NH_2_ (25.0 mg, 0.067 mmol) and *N,N*-diisopropylethylamine (DIPEA; 24.2 $\mu$L, 0.146 mmol). This was followed by the drop-wise addition of O-(benzotriazol-yl)-*N,N,N’N’*-tetrauronium hexafluorophosphate (HBTU, 27.837 mg, 0.073 mmol) dissolved in minimum DMF. The reaction mixture was left for 4 h at room temperature and monitored using a TLC. At the end of the reaction, the mixture was diluted with 100 mL of dichloromethane (DCM) and thoroughly washed with water (3 x 100 mL), followed by further extractions of the combined aqueous fractions using DCM (2 x 100 mL). The organic layers were washed with brine (200 mL), dried using anhydrous Na_2_SO_4_, filtered and evaporated to dryness. The crude product was separated using a silica gel preparative TLC plate using ethyl acetate: methanol (90:10 v/v) system to afford compound **1** as a pale yellow oily product (45 mg, 84.25%).

^1^H NMR (300 MHz, CD_2_Cl_3_-*d*): 7.88 (m, 2H, C=CH), 7.5-7.04 (m, 8H, Ar-H), 6.28 (d, 1H, CH=CH, J=11.7), 5.86 (d, 1H, CH=CH, J=11.7), 4.77 (s, 2H), 4.55 (s, 2H), 4.38 (s, 1H), 4.19 (s, 1H), 3.59-3.19 (m, 10H), 2.83 (brs, 4H), 2.75 (brs, 3H), 2.68 (brs, 1H), 2.12 (m, 2H), 1.63-1.32 (m, 6H).

HRMS (ESI): calc for C_39_H_46_Cl_2_N_5_O_7_S [M+H]^+^: 798.2495 and C_39_H_45_Cl_2_N_5_O_7_SNa [M +Na]^+^:820.2314, found 798.2465 and 820.2292.

**1.2 HO-EF24-Biotin (2)**

The procedure to synthesize **2** was similar to that used for the synthesis of **1.** Summarily, using EF24-OH (100 mg, 0.319 mmol) biotin (78.03 mg, 0.319 mmol), DIPEA (115.70 $\mu$L, 0.703 mmol) and HBTU (133.2 mg, 0.351 mmol) in 4 mL anhydrous DMF, compound **2** was separated as white solid (112 mg, 65.11%).

^1^H NMR (300 MHz, CDCl_3_-*d_3_*): $\delta$ 8.00 (s, DMF), 7.45-6.97 (m, 8H, Ar-H), 6.71 (s, 2H C=CH) 4.93 (s, 4H), 4.43 (m, 1H), 4.17 (m, 1H) 3.04 (m 1H) 2.92 (brs, DMF), 2.86 (brs, DMF), 2.77 (brs, 2H), 2.12 (m, 2H), 1.67-1.34 (m, 6H). HRMS (ESI): calc for C_29_H_31_F_2_N_3_O_3_SNa [M +Na]^+^:562.1952, found 562.1985.

**1.3 Biotin-O-EF24 (5)**

Synthesis of compound **5** involves three steps: a) Boc-protection of the reactive sec-NH group, b) esterification of -COOH group in biotin with free -OH group of EF24-OH, and c) boc- deprotection.

*HO-EF24-Boc* (**3**): To 6 mL anhydrous methanol were added HO-EF24 (215 mg, 0.687 mmol), followed by DIPEA (2.061 mmol). The reaction mixture was stirred at room temperature for 15 min. Di-tert-butyl dicarbonate (Boc_2_O) was added to the reaction mixture under nitrogen atmosphere and refluxed at 80 ^o^C for 24 h. The reaction mixture was diluted with DCM and then washed with saturated NaHCO_3_ solution (2 x 200 mL), followed with water (300 mL). The product was dried with anhydrous Na_2_SO_4_ and evaporated to dryness in vacuo. Silica gel Prep TLC plate was used to separate the protected compound using ethyl acetate to afford **3** as pale yellow solid (250 mg, 88.09%).

^1^H NMR (300 MHz, CDCl_3_-*d_3_*): $\delta$ 7.45-7.01(m, 8H Ar-H), 6.70 (s, 2H C=CH) 4.89 (s, 1H), 4.30 (s, 4H), 1.21 (s, 9H). ^13^C NMR (75 MHz, CDCl_3_-*d_3_*): $\delta$ 161.42, 158.45, 154.58, 131.09, 129.03, 123.75, 115.51, 114.96, 80.16, 75.26, 29.92, 27.44.

HRMS (ESI): calc for C_24_H_25_F_2_NO_3_Na [M +Na]^+^:436.1700, found 436.1641.

*Biotin-O-EF24-Boc* (**4**): To 4 mL of anhydrous DMF were added Biotin (118.711 mg, 0.4858 mmol), dimethylaminopyridine (26.98 mg, 0.221 mmol), and HO-EF24-Boc (**3**) (182.5 mg, 0.442 mmol). The reaction mixture was cooled to 0 ^o^C and dicyclohexylcarbodiimide (109.3 mg, 0.531 mmol) in minimum amount of DMF was added drop-wise. The reaction mixture was stirred at room temperature overnight. The crude extract was diluted with DCM (200 mL), washed with 1 M HCl (50 mL), followed by washing with saturated NaHCO_3_ and then water. The organic layer was dried over anhydrous Na_2_SO_4_, filtered and evaporated to dryness. The crude mixture was separated using prep TLC using ethylacetate:methanol (96:4 v/v) to obtain protected product (**4**) as white solid (210 mg, 81.46 %).

HRMS (ESI): calc for C_34_H_39_F_2_N_3_O_5_SNa [M +Na]^+^:662.2476, found 662.2542.

*Biotin-O-EF24* (**5**): Boc-protection was removed by adding 3 drops of trifluoroacetic acid to a solution of **4** in DMF and stirring for 3 h. The solvent was removed using high vacuum to give a white solid of Biotin-O-EF24 (**5**) which was used without further purification.

^1^H NMR (300 MHz, CDCl_3_-*d_3_*): $\delta$ 7.51-7.05 (m, 8H, Ar-H), 6.76 (s, 2H), 4.47 (m, 4H), 4.31 (m, 2H), 4.06 (m, 1H), 3.57 (m, 1H), 3.31 (brs, 2H) 2.38 (m, 2H), 1.88-123 (m, 6H)

**1.4 EF24-PEG_8_-Biotin** (**7**)

Compound **7** was synthesized via two steps, viz; reaction EF24 with succinic anhydride to afford compound **6** and a reaction of **6** with Biotin-PEG_8_-amine.

Compound **6** was synthesized as reported in literature (Bioorg Med Chem. 2010 Aug 15; 18(16): 6109-20). The product was purified using Prep TLC plate instead of column chromatography. Summarily, succinic anhydride (80.4 mg, 0.802 mmol), DIPEA (264 $\mu$L, 1.607 mmol) and EF24 (250 mg, 0.802 mmol) in 10 mL of DCM afforded compound **6** as yellow oil (250 mg, 75.82%). All characteristics matched with reported data.

EF24-PEG_8_-Biotin (**7**) was synthesized by a reaction of compound **6** with biotin-PEG_8_-amine using a method similar to the one described above for compound **1**. Separation was effected on Sephadex LH-20 column with metanol as the eluent to afford **7** as yellow oil (50 mg, 84%).

^1^H NMR (300 MHz, CDCl_3_-*d_3_*): $\delta$ 7.89 (s, 2H amide N-H), 7.50-7.03 (m, 8H, Ar-H), 6.63 (s, 1H C=CH), 6.47 (s, 1H C=CH), 5.97 (s, 2H, Biotin N-H), 5.28 (s, 1H), 4.78 (s, 2H), 4.63 (s, 2H), 4.47 (m, 1H Biotin), 4.27 (s, 1H Biotin), 3.73-3.25 (m, -CH_2_-CH_2_O, complex), 2.52-2.36 (m, 4H), 2.16 (m, 2H), 1.38-1.77 (m, 6H)

HRMS (ESI): calc for C_53_H75F_2_N_5_O_14_SNa [M +Na]^+^:1075.4897, found 1098.5227.

**2 Proteasome inhibition by CLEFMA and EF24**

**Figure S1:** Dose-response curves for calculation of IC50 values of CLEFMA and EF24 for inhibition of trypsin-like and chymotrypsin-like proteasome activities.





**3 Table S1**

Identity of constituent proteins in CLEFMA+19S mixture subjected to 2D-gel electrophoresis and LC-MS/MS. Mass spectroscopy report is from Clarkson University Protein Core Facility (courtesy: Kendrick Labs, Madison, WI) showing analyses of four spots identified in Fig. 7 of the main text.

**4 Table S2**

Constituent protein subunits of the 19S regulatory particle. Highlighted subunits are reasonably close to the 43kDa protein which interacted with CLFMA and EF24.

| **19S Regulatory Particle Subunits** | **Mol Mass** | **Gene** | **Common name** |
| --- | --- | --- | --- |
| 26S proteasome non-ATPase regulatory subunit 1 | 105.8 | PSMD1 | RPN2 |
| 26S proteasome non-ATPase regulatory subunit 2 | 100.2 | PSMD2 | RPN1 |
| 26S proteasome non-ATPase regulatory subunit 3 | 60.98 | PSMD3 | RPN3 |
| 26S proteasome non-ATPase regulatory subunit 4 | 40.74 | PSMD4 | RPN10 or S5A |
| 26S proteasome non-ATPase regulatory subunit 5 | 56.2 | PSMD5 | S5B |
| 26S proteasome non-ATPase regulatory subunit 6 | 45.5 | PSMD6 | RPN7 or S10 |
| 26S proteasome non-ATPase regulatory subunit 7 | 37.02 | PSMD7 | RPN8 or S12 |
| 26S proteasome non-ATPase regulatory subunit 8 | 39.61 | PSMD8 | RPN12 or S14 |
| 26S proteasome non-ATPase regulatory subunit 9 | 24.68 | PSMD9 | -- |
| 26S proteasome non-ATPase regulatory subunit 10 | 24.43 | PSMD10 | RPN4 |
| 26S proteasome non-ATPase regulatory subunit 11 | 47.46 | PSMD11 | RPN6 |
| 26S proteasome non-ATPase regulatory subunit 12 | 52.9 | PSMD12 | RPN5 |
| 26S proteasome non-ATPase regulatory subunit 13 | 42.95 | PSMD13 | RPN9 |
| Proteasomal ubiquitin receptor ADRM1 | 42.15 | ARM1 | RPN13 |
| 26S proteasome regulatory subunit 4 | 49.19 | PSMC1 | RPT2 |
| 26S proteasome regulatory subunit 6B | 47.37 | PSMC4 | RPT3 |
| 26S proteasome regulatory subunit 6A | 49.2 | PSMC3 | RPT5 |
| 26S proteasome regulatory subunit 7 | 48.6 | PSMC2 | RPT1 |
| 26S proteasome regulatory subunit 8 | 45.63 | PSMC5 | RPT6 |
| 26S proteasome regulatory subunit 10B | 44.17 | PSMC6 | RPT4 |
